# Consolidation of the Buffalo Medical Schools

**Published:** 1898-07

**Authors:** 


### CONSOLIDATION OF THE BUFFALO MEDICAL SCHOOLS.

IN AN appropriate place announcement is made of the union of Niagara University medical college with that of the Buffalo University medical school. There was undoubtedly some surprise among the friends of both colleges when the announcement appeared in the newspapers, and perhaps it occasioned regret on the part of some friends of Niagara, especially its alumni. On this account perhaps a few words on the subject in these columns may not be deemed out of place.

When the medical department of Niagara University was organised fifteen years ago it was believed that there was room for such an institution with advanced standards, which should include higher preliminaries, longer college terms and separate final examinations. These were all adopted by Niagara and the history of the college shows that it was not organised in vain, nor has its example proved futile. It has served to point the way to a better state of affairs in medical education not only in this state but all over the country.

At the present time it is required in this state on the part of a medical student, that he shall possess an English education equivalent to that of a graduate of a high school before he can enter upon the study of medicine. He must then attend a medical college for four years, after which, if qualified, he may receive a diploma. This, however, will not entitle him to practise; for he must now submit to an examination by the state, which if successfully met will entitle him to a license.

When Niagara was organised in 1883, no preliminaries were necessary, except such as each medical school might see fit to impose, two years was a legal college course, and a medical diploma, no matter how obtained we had almost said, conferred a right to practise. Who shall gainsay the statement that the bold stand taken by the faculty of Niagara in 1883 has not contributed to the conditions prevailing in 1898? Dare anyone assert that Niagara as a pioneer in the movement toward higher medical education in this state is not entitled to a high measure of praise? If the school in view of the changed conditions herein referred to has apparently outlived its usefulness, is there any dishonor in yielding up its individuality? When the great volunteer army of the civil war had conquered a peace it disbanded and returned to civil pursuits.

Let the alumni of Niagara always feel pride in the honorable part their alma mater bore in the contest for better education, and if a feeling of regret cannot be repressed at its union with Buffalo, thus sinking its identity in a measure, let it always be remembered that its fifteen years of collegiate life can be pointed to as an era that adorns the brightest page of medical history in this country—that of reform in medical education. All honor, too, to the faculty that made sacrifices to accomplish such results and that further sacrificed its separate existence as a teaching body to the spirit of the age which has announced to the people with all the forcefulness of a Delphic oracle "There are too many medical colleges."

---

The *New York Journal* added to the medical literature of the country a week or two ago by publishing reports of two discoveries. One was a tuberculosis "cure" credited to Dr. Murphy, of Chicago, the inventor of the Murphy button; the other Prof. Koch's discovery that the malaria germ was transmitted by the mosquito. In justice to Dr. Murphy it should be said that the word "cure" is used by the *Journal* and not by him. These reports are illustrated with newspaper sketches of the germs of tuberculosis and malaria which are amusing evidence of the inaccuracy of newspaper science. The sketches of the tubercle bacillus look more like a flock of wild geese making their fall flight than anything else, while as for the malaria germs, the sketch has a sort of a Good Friday flavor, the illustrations appearing more like a series of hot cross buns than pictures of plasmodia or crescents. Newspapers are great factors for good and are admirable public educators, but they are as yet rather astigmatised in looking at scientific matters. Their well-intentioned efforts and their accomplishments do not always match.

---

ACCORDING to the advertisement of one of the New York department stores, printed in the *New York Herald* a short time ago, the Murphy button seems doomed to disuse. The advertisement, after speaking of the many useful articles for sale for wedding presents, gravely announced a fine assortment of "handsome glass bowels." Just imagine the operative procedures of the future when it will only be necessary to send in an order to the department store for some "handsome glass bowels" and put 'em in. Then, indeed, there will come sad days for the Murphy button and its merry click will be silenced forever.

---

Richard Harding Davis took his pen in hand awhile ago and wrote for the *New York Herald* an article describing a laparatomy performed on the cruiser New York. It was thrilling to the lay mind; it was bloody; it was a lurid picture, painted by a fleeting pen in the hands of an accomplished but over-enthusiastic and highly imaginative novelist. Mr. Davis said, in describing events after the operation, that the patient lying on a cot, opened his eyes and saw the surgeons standing about him with arms covered with blood up to the elbows. Then he added that the operating table "dripped with blood." Surely these be bleeding war times, but we hope the New York recovered.

---

The Journal learns on what may be considered very good authority that Dr. Wende's efforts in behalf of the schools and school children will be crowned with success in the near future.

---

On account of the pressure upon our columns this month we have been compelled to omit the publication of the chapter on medical history in Erie county. It will, however, be resumed in August and continued without intermission until completed.

